# 1002. Case Study. Achieving Successful Closure of Necrotizing Soft Tissue Infection Wound with Autograft and Bioresorbable Antimicrobial Matrix (A Case Study)

**DOI:** 10.1093/jbcr/irag033.190

**Published:** 2026-04-06

**Authors:** Carmen E Flores

**Affiliations:** Leon S. Peters Burn Center, Las Vegas, NV

## Abstract

**Patient Presentation (age range, injury details, relevant history):**

A 60-year-old paraplegic male with a history of stroke, hypertension, and iatrogenic spinal cord injury with cauda equina sequelae was transferred to our facility in septic shock nine days post-injury. He had undergone two prior excisions at an outside hospital for necrotizing soft tissue infection (NSTI) involving the lower upper extremity and right thigh before direct transfer to the intensive care unit.

**Clinical Challenges:**

NSTI wounds pose significant challenges due to rapid tissue necrosis, systemic toxicity, and frequent need for repeated surgical intervention. Use of a fully synthetic, bioresorbable antimicrobial matrix has demonstrated potential in reducing infection risk in challenging wound populations. This report describes the successful use of such a matrix to achieve definitive wound closure in a patient with extensive infection.

**Management Approach:**

The wound was excised to bone, elbow joint space, tendon, and muscle. Wound bed preparation included negative pressure wound therapy (NPWT) and an electrospun fiber matrix, combined with nutritional support and physical/occupational therapy to optimize healing. Once deep structures were no longer exposed, split-thickness skin grafting and epidermal autograft spray were performed on hospital day 46. The bioresorbable antimicrobial matrix was applied directly over the autografts before NPWT. The matrix was also applied to the donor thigh site after epidermal autograft spray, followed by NPWT and additional coverage with a native collagen wound dressing on post-operative day four. Secondary dressings included a nonadherent contact layer and hypochlorous acid wet-to-moist dressing.

**Outcomes:**

By post-operative day four, the graft was unoccluded and 90% epithelialized. Complete wound closure was observed by post-operative day five and donor site healing by day seven. By day 10, both donor sites were fully healed, and the wound demonstrated excellent functional outcomes without contracture. No recurrent infection was observed beyond the initial presentation.

**Lessons Learned:**

Use of a bioresorbable antimicrobial matrix contributed to timely, uncomplicated closure of NSTI and donor site wounds in a septic patient. The case demonstrates feasibility of incorporating the matrix into institutional standard of care, given its compatibility with both traditional modalities (NPWT) and newer adjuncts (epidermal autograft spray).

**Applicability to Practice:**

This case highlights the potential role of bioresorbable antimicrobial matrices as a valuable adjunct for achieving closure of extensively infected wounds in complex patients.

